# The Distinct Function of p21^Waf1/Cip1^ With p16^Ink4a^ in Modulating Aging Phenotypes of Werner Syndrome by Affecting Tissue Homeostasis

**DOI:** 10.3389/fgene.2021.597566

**Published:** 2021-02-05

**Authors:** Yongjin Zhang, Chihao Shao, Haili Li, Kun Wu, Lixin Gong, Quan Zheng, Juhua Dan, Shuting Jia, Xiaodan Tang, Xiaoming Wu, Ying Luo

**Affiliations:** ^1^Laboratory of Molecular Genetics of Aging & Tumor, Medical School, Kunming University of Science and Technology, Kunming, China; ^2^Guizhou Provincial Key Laboratory of Pathogenesis & Drug Development on Common Chronic Diseases, School of Basic Medicine, Guizhou Medical University, Guiyang, China; ^3^Yunnan Provincial Institute of Digestive Disease, Department of Gastroenterology, First People’s Hospital of Yunnan Province, Kunming, China

**Keywords:** p21Waf1/Cip1, p16INK4a, aging, tissue homeostasis, Werner syndrome

## Abstract

Human Werner syndrome (WS) is an autosomal recessive progeria disease. A mouse model of WS manifests the disease through telomere dysfunction-induced aging phenotypes, which might result from cell cycle control and cellular senescence. Both p21^Waf1/Cip1^ (p21, encoded by the *Cdkn1a* gene) and p16^Ink4a^ (p16, encoded by the *Ink4a* gene) are cell cycle inhibitors and are involved in regulating two key pathways of cellular senescence. To test the effect of p21 and p16 deficiencies in WS, we crossed WS mice (DKO) with *p21*^–/–^ or *p16*^–/–^ mice to construct triple knockout (p21-TKO or p16-TKO) mice. By studying the survival curve, bone density, regenerative tissue (testis), and stem cell capacity (intestine), we surprisingly found that p21-TKO mice displayed accelerated premature aging compared with DKO mice, while p16-TKO mice showed attenuation of the aging phenotypes. The incidence of apoptosis and cellular senescence were upregulated in p21-TKO mice tissue and downregulated in p16-TKO mice. Surprisingly, cellular proliferation in p21-TKO mice tissue was also upregulated, and the p21-TKO mice did not show telomere shortening compared with age-matched DKO mice, although p16-TKO mice displayed obvious enhancement of telomere lengthening. Consistent with these phenotypes, the SIRT1-PGC1 pathway was upregulated in p16-TKO but downregulated in p21-TKO compared with DKO mouse embryo fibroblasts (MEFs). However, the DNA damage response pathway was highly activated in p21-TKO, but rescued in p16-TKO, compared with DKO MEFs. These data suggest that p21 protected the stem cell reservoir by regulating cellular proliferation and turnover at a proper rate and that p21 loss in WS activated fairly severe DNA damage responses (DDR), which might cause an abnormal increase in tissue homeostasis. On the other hand, p16 promoted cellular senescence by inhibiting cellular proliferation, and p16 deficiency released this barrier signal without causing severe DDR.

## Introduction

Knowledge of the aging mechanism is essential for understanding aging-related diseases, such as tumorigenesis and organ degeneration. At the organismal level, aging demonstrates typical phenotypes such as degenerative organ function and dysfunction of hormone homeostasis, which results in the development of cataracts, osteoporosis, diabetes, neurodegenerative diseases, cardiovascular diseases, and tumorigenesis. At the cellular level, phenotypes of cellular senescence include a decrease in stem cell capacity, abnormal cell-cell communication, abnormal mitochondrial function, chromosome instability, dysfunction of protein homeostasis, telomere dysfunction, etc. (Lopez-Otin et al., 2013). Telomere dysfunction, including telomere attrition, telomere DNA damage, and abnormal telomere structure, is the key player in cell replicative senescence. It might also account for organ aging. The well-established biomarkers for cellular senescence are telomere dysfunction, elevated p16^Ink4a^ levels, persistent DNA damage responses (DDR), senescence-associated secretion phenotype (SASP), senescence-associated heterochromatin foci (SAHF), positive SA-β-gal staining, downregulation of lamin B1, etc. (He and Sharpless, 2017).

The progeroid diseases derived from telomere dysfunction provide a perfect model system for aging research. Werner syndrome (WS) is a rare autosomal recessive genetic disease. The premature aging phenotypes of WS include short average lifespan (46–48 years), early onset atherosclerosis, cataracts, osteoporosis, type II diabetes mellitus, and an elevated incidence of soft tissue sarcoma (Epstein et al., 1966). The fibroblasts of WS patients have been isolated and revealed a high instability of chromosomes in WS cells, known as mosaic chromosomes (Salk et al., 1981). Further study revealed that Wrn gene mutation is the cause of WS. The Wrn gene encodes the WRN protein and is one of the members of the DNA helicase RecQ family. Wrn protein is involved in DNA replication, recombination and DNA damage repair and thus plays an essential role in maintaining chromosome stability (Yu et al., 1996).

However, mice with a Wrn deletion did not develop any obvious aging phenotypes (Lebel and Leder, 1998). Fibroblast and B-lymphoblastoid cell lines derived from WS patients exhibited accelerated rates of telomere shortening compared to age-matched control cells (Lebel and Leder, 1998), and senescence of fibroblasts from WS patients could be rescued by the overexpression of telomerase (Wyllie et al., 2000). These data suggested that telomere dysfunction might play an essential role in the manifestation of the WS phenotypes. In contrast to humans, adult mouse cells still maintain telomerase activity, and the chromosomes of mouse cells contain long telomeres, which might prevent progeria phenotypes in Wrn-deficient mice. Two laboratories independently generated mice with both telomerase and *Wrn* deficiencies. The late generations (G4-6) of m*Terc^–/–^Wrn^–/–^*mice exhibited many of the clinical features observed in WS patients, including early onset of wound-healing defects, osteoporosis with skeletal fractures, hypogonadism, cataract formation, type II diabetes and short life span. However, G3 m*Terc^–/–^Wrn^–/–^*mice with longer telomeres did not show these premature aging phenotypes, suggesting that loss of Wrn and in the setting of short telomere is necessary for the manifestation of WS in mice. At the cellular level, Wrn deficiency and telomere dysfunction cooperated to accelerate telomere shortening, resulting in accelerated cellular senescence. Elevated telomere–telomere recombination in these cells promotes escape from senescence and engagement of tumorigenesis through alternative lengthening of the telomere (ALT) (Laud et al., 2005).

It has been well accepted that the DDR signaling pathway regulated by tumor suppressors p53 and Rb is key for cellular senescence (Ben-Porath and Weinberg, 2005). In this signaling cascade, p16^Ink4a^ (p16) and p21^Waf1/Cip1^ (p21) directly regulate the cell cycle progress by inhibiting cyclin/Cdks; thus, p16 and p21 are believed to be the key effectors for cellular senescence. In recent years, many studies have been conducted to understand the function of p16 or p21 in the aging process or tumorigenesis. It has been demonstrated that in the absence of p16, the repopulating defects of hematopoietic stem cells (HSCs) have been reversed and the survival of animals with successive bone marrow transplantation has been improved (Janzen et al., 2006). Compared with wild-type mice, p16-deficient mice have a significantly smaller decline in subventricular zone proliferation, olfactory bulb neurogenesis, and the frequency and self-renewal potential of multipotent progenitors (Molofsky et al., 2006). Another study showed that p16 constrained pancreatic islet proliferation and regeneration in an age-dependent manner. They found that islet proliferation was relatively increased in p16-deficient mice of old age, and the mice lacking p16 demonstrated enhanced islet proliferation and survival after b-cell ablation (Krishnamurthy et al., 2006). Together, these data suggest that p16 limits the regenerative capacity of tissue stem cells with aging, and the inhibition of p16 may rescue stem cell aging and improve injury repair in aging tissue.

A series of studies were conducted to investigate the role of p16 based on the BubR1 progeroid mouse model. Targeting the clearance of p16-positive senescent cells in BubR1 progeroid mice revealed that the life-long removal of p16-expressing cells delayed the onset of aging phenotypes in adipose tissue, skeletal muscles, and eyes. Late-life clearance also attenuated the progression of already established age-related disorders (Baker et al., 2011). Further study showed that senescent fat progenitors secrete activin A and directly inhibit adipogenesis. Targeting the clearance of senescent cells from aged mice could reduce circulating activin A, rescue fat loss, and enhance adipogenesis (Xu et al., 2015).

The senescent mouse embryo fibroblasts (MEFs) derived from WS mice could escape senescence and form immortalized cell lines (Laud et al., 2005). Our previous data showed that the p16 expression level of these immortalized cell lines has been downregulated, regardless of whether they are tumorigenic or nontumorigenic (Wu et al., 2012). The knockout of p16 in the background of telomere dysfunction could rescue telomere DNA damage-induced senescence (Zhang et al., 2012). These data suggest that p16 deficiency could rescue telomere dysfunction-induced senescence.

The role of p21 in the aging process has not been well studied. It has been demonstrated that loss of p21 function did not show any acceleration of tumorigenesis during the first year of life (Lebel et al., 2001). It has also been shown that knocking out p21 could extend the lifespan of mice with a deficiency of telomerase RNA template (*Terc*^–/–^), without increasing chromosome instability and tumorigenesis. The deficiency of p21 also improved the HSC function and intestinal epithelial homeostasis in *Terc* knockout mice and enhanced the HSC self-renewal capacity and the repopulation function (Choudhury et al., 2007). These data suggest that p21 could rescue the aging phenotype of *Terc* knockout mice. Consistent with this, our data also showed that in MEFs with telomere dysfunction induced by TRF2^ΔBΔM^ overexpression, p21 deficiency could rescue cellular senescence without increasing tumorigenesis. However, our data also showed that when we strengthened telomere dysfunction with Wrn deficiency and TRF2^ΔBΔM^ overexpression (*Wrn*^–/–^TRF2^ΔBΔM^), p21 deficiency with this severe telomere dysfunction resulted in tumorigenesis. Our data suggest that p21 deficiency plays a distinct role in the response to different degrees of telomere dysfunction, reversing aging or tumorigenesis (Si et al., 2018).

To clarify the function of p21 or p16 in modulating aging phenotypes in the background of WS, we crossed the WS mice with *p21*^–/–^ or *p16*^–/–^ mice and obtained triple knockout mice *mTer*^–/–^*Wrn*^–/–^*p21*^–/–^ (p21-TKO) or *mTer*^–/–^*Wrn*^–/–^*p16*^–/–^ (p16-TKO). The mice were then bred generation by generation and G2, G3, G4, and G5 TKO mice were obtained. We aim to explore the effect of p21 and p16 in modulating the WS aging phenotype and provide targets for anti-aging drug screening.

## Results

### p21 Deficiency Shortened the Llifespan of Werner Syndrome Mice, While p16 Deficiency Prolonged It

**FIGURE 1:**
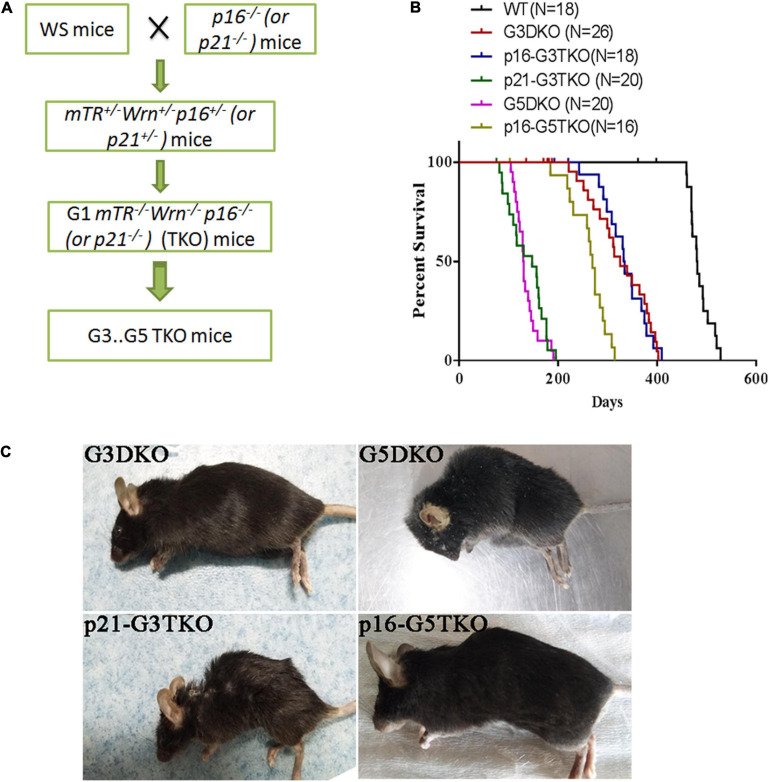
The generation and lifespan of p16/p21-TKO mice. **(A)** The breeding strategy for generating p16/p21TKO mice. The *p16*^– /–^ or *p21*^– /–^ mice were crossed with WS mice and G1TKO (*G1mTR*^– /–^ *Wrn*^– /–^ *p21*^– /–^ or *G1mTR*^– /–^ *Wrn*^– /–^ *p16*^– /–^ ) mice were obtained. Then, the mice were bred generation by generation to obtain G2, G3, G4, G5 TKO mice. **(B)** Kaplan-Meier survival curves of G3DKO, p16-G3TKO, p21-G3TKO, G5DKO, and p16-G5TKO mice. For G3DKO, p21 deficiency shortened the lifespan (*p* < 0.001), while p16 deficiency slightly increased the lifespan (*p* = 0.0018). For G5DKO, p16 deficiency dramatically prolonged the lifespan (*p* < 0.001). Because an accelerated aging phenotype occurred in p21-TKO, mice with this genotype could only be bred to G3TKO. **(C)** The mouse appearance and body size also showed that p21 deficiency accelerated the aging phenotypes of WS, while p16 deficiency rescued it.

To investigate the role of p21 or p16 in the aging process of WS, we crossed the WS mice with *p21*^–/–^ or *p16*^–/–^ mice and obtained the first generation (G1) triple knockout mice *mTer*^–/–^*Wrn*^–/–^*p21*^–/–^ (p21-TKO) or *mTer*^–/–^*Wrn*^–/–^*p16*^–/–^ (p16-TKO). The mice were then inbred generation by generation and G2, G3, G4, and G5 TKO mice were obtained (Figure 1A). After collecting the survival data for mice with different genotypes, we plotted the Kaplan-Meier survival curves for mice with different genotypes (Figure 1B). When WS mice were inbred to the third generation (G3DKO), the telomere was a medium length, and the mice had not yet displayed obvious aging phenotypes. The average lifespan for G3DKO mice was 281 ± 24 days. However, with p21 deficiency, the average lifespan for p21-G3DKO mice was decreased to 136 ± 9 days. Interestingly, with p16 deficiency, the average lifespan for p16-G3TKO mice was increased to 334 ± 16 days (Figure 1B). These data revealed that p21 deficiency dramatically shortened the lifespan of WS (*p* < 0.001). Surprisingly, the lifespan of p21-G3TKO mice was similar to the lifespan of G5DKO mice, in which the telomere was severely shortened, and the average lifespan of G5DKO mice was 136 ± 6 days. Therefore, we could not obtain the generation of a mouse later than p21-G3TKO. On the other hand, even though not very obvious, p16 deficiency started to rescue the WS aging process when telomere attrition started to occur at G3DKO(*p* = 0.0018). The elongation of lifespan by p16 deficiency became dramatic when telomeres became severely attrited in G5DKO mice with p16 deficiency (*p* < 0.001). The average lifespan for p16-G5TKO mice was increased to 236 ± 19 days (Figure 1B).

Furthermore, the appearance of the mice also showed dramatically different effects of p21 or p16 deficiency in regulating WS aging phenotypes. The appearances of the p21-G3TKO and G5DKO mice were similar; they were small in size and had obvious kyphosis compared to the age-matched G3DKO mice. However, p16-G5TKO mice appeared similar to G3DKO mice by appearance (Figure 1B).

Together, these data strongly suggested that p21 deficiency accelerated the WS aging process, while p16 deficiency rescued it. Most importantly, we have not observed any tumorigenesis events in p16-G5TKO mice thus far.

### p21 Deficiency Accelerated the Degeneration of Organs in Werner Syndrome Mice During the Aging Process, While p16 Deficiency Rescued It

To further dissect the aging phenotypes of WS affected by p21 or p16 deficiency, we investigated the degeneration of organs that were most affected in aging processes, such as bone, testis, intestine, etc.

**FIGURE 2:**
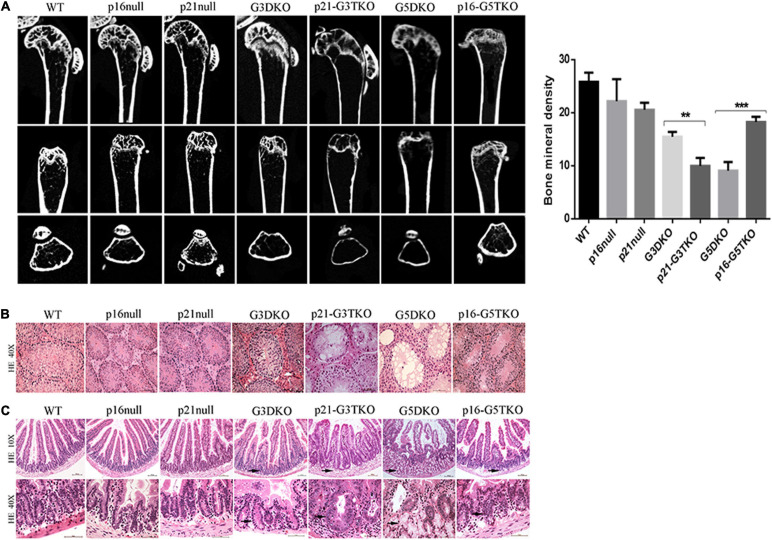
Tissue aging phenotypes in p16/p21-TKO mice. **(A)** The bone density measured by miroCT. Micro CT images of distal femoral metaphysis from mice with different genotypes. The femurs are shown from the coronal **(upper panel)**, sagittal **(middle panel)**, and transverse **(lower panel)** planes, respectively. Quantification of the bone density and statistical analysis. **p = 0.01,***p < 0.01. **(B)** HE staining of testes. The testes from p21-G3TKO and G5DKO mice were undergoing severe degeneration, showing a lack of spermatogenesis in seminiferous tubules. The deficiency of p16 in G5DKO rescued this testis degeneration phenotype. **(C)** HE staining of intestines. The intestines from p21-G3TKO and G5DKO mice were undergoing severe degeneration, showing an irregular intestinal villi structure, especially in crypt structures (10×, arrows pointed). At higher magnification, the crypts of p21-G3TKO mice seemed to be swollen and enlarged in size, while the crypts of G5TKO mice were empty due to loss of cells (40X, arrows pointed). p16 deficiency rescued the loss of crypt structure in G5DKO.

The bone density detected by microCT revealed that the loss of bone mass in the spines and femurs of p21-G3TKO mice was dramatically increased compared with age-matched G3TKO mice, and this was not observed in mice with only p21 deficiency (Figure 2A, p21 null, G3DKO, p21-G3TKO). We observed similar bone loss in age-matched G5DKO mice; however, this bone loss was rescued by p16 deficiency (Figure 2A, p16 null, G5DKO, p16-G5TKO).

HE staining revealed that the testes from p21-G3TKO and G5DKO mice were undergoing severe degeneration, showing a lack of spermatogenesis in seminiferous tubules, which was not observed in age-matched G3DKO mice. The deficiency of p16 in G5DKO rescued this testis degeneration phenotype (Figure 2B, p21-G3TKO, G5DKO). The intestines from p21-G3TKO and G5DKO mice also underwent severe degeneration, showing an irregular intestinal villi structure, especially in crypt structures (Figure 2C, 10X, arrows pointed). At higher magnification, we observed that the crypts of p21-G3TKO mice seemed to be swollen and enlarged in size, while the crypts of G5TKO mice were empty due to loss of cells. Here, again, p16 deficiency rescued the loss of crypt structure in G5DKO (Figure 2C, 40X, arrows pointed).

As we know, the crypt structure of the intestine is the compartment for intestinal stem cells, and spermatogenesis is also related to the stemness capacity of an organism. We wondered whether p21 or p16 deficiency actually affected the senescence and stemness maintenance of proliferative tissue in WS mice.

**FIGURE 3:**
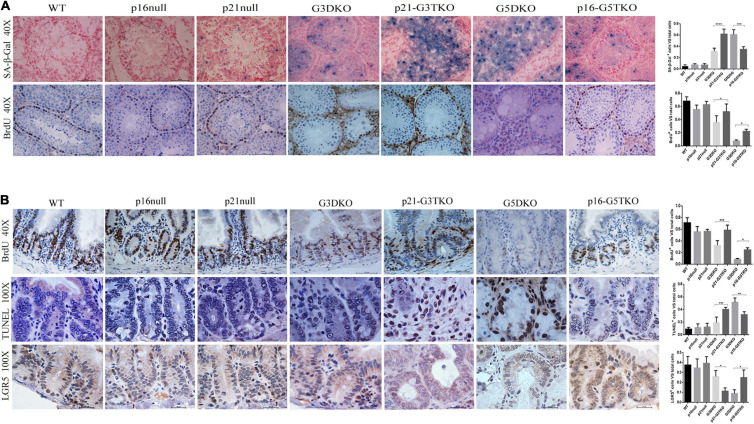
Cellular senescence, proliferation, and apoptosis occurred in tissues from p16/p21-TKO mice. **(A)** The cellular senescence and proliferation of testes detected by SA-β-Gal staining and BrdU incorporation. The p21-G3TKO and G5DKO testes showed comparable amounts of cellular senescence. However, p21-G3TKO testes maintained a high level of cellular proliferation, which was decreased dramatically in G5DKO testes and rescued in p16-TKO testes. Quantification of SA-β-Gal staining and BrdU incorporation and statistical analysis. *p < 0.05, **p < 0.01, ****p < 0.001. **(B)** The cellular proliferation, apoptosis, and stem cell capacity of intestinal villi detected by BrdU incorporation, TUNEL assay, and LGR5 immunostaining. Compared with G3DKO, the p21-G3DKO intestine showed increased cellular proliferation and apoptosis but decreased stem cell capacity. The p16-TKO intestine showed rescued cellular proliferation and stem cell capacity and decreased apoptosis compared with G5DKO intestines. Quantification of the cellular proliferation, apoptosis, and stem cell capacity and statistical analysis. *p < 0.05, **p < 0.01, ****p < 0.001.

To verify this, we applied SA-β-Gal staining and BrdU incorporation to the tissues of interest, together with immunostaining for stem cell markers. With SA-β-Gal staining, increased senescent cells were observed in the testes of p21-G3TKO and G5DKO mice compared with G3TKO mice. Again, p16 deficiency decreased the level of senescence in G5DKO mice (Figure 3A, SA-β-Gal, G3DKO, p21-G3TKO, G5DKO, p16-G5TKO). Interestingly, we observed a slight increase in senescence in the testis with only p21 deficiency compared with the wild-type testis or testis with only p16 deficiency (Figure 3A, SA-β-Gal, WT, p16 null, p21 null). Surprisingly, even with increased senescence levels in the testes of p21 null mice, the proliferation level displayed with BrdU incorporation did not decrease (Figure 3A, BrdU, WT, p16 null, p21 null). The increase of both senescence and proliferation by p21 deficiency became more obvious in the testes of p21-G3TKO mice compared with G3DKO mice, suggesting that a correlation of senescence and proliferation was prohibited by p21 function (Figure 3A, G3DKO, p21-G3TKO). We did not observe this phenomenon in p16-deficient mice. Compared with G5DKO, the testes from p16-G5TKO mice displayed decreased senescence levels and reasonably increased proliferation levels (Figure 3A, G5DKO, p16-G5TKO). These data suggested the distinct function of p21 and p16 in regulating cellular proliferation and senescence.

Since intestinal villi undergo stem cell renewal and cellular proliferation processes consistently, we also investigated the cellular turnover in intestinal villi by BrdU incorporation. The data showed that compared with G3DKO mice, the intestines from p21-G3TKO mice showed increased BrdU incorporation, suggesting an increased proliferation rate derived from p21 deficiency (Figure 3B, BrdU, G3DKO, p21-G3TKO), which was also observed in p16 deficiency (Figure 3B, BrdU, G5DKO, p16-G5TKO). By TUNEL assay, we detected the apoptosis level in the intestines, and the data showed that p21 deficiency resulted in dramatically increased apoptosis in G3DKO. The apoptosis level in p21-G3TKO is comparable to that in G5DKO (Figure 3B, TUNEL, G3DKO, p21-G3TKO, G5DKO). However, p16 deficiency successfully reduced apoptosis in G5DKO mice (Figure 3B, TUNEL, G5DKO, p16-G5TKO).

Since the crypts are the stem cell compartments for the intestines, we further applied immunostaining for the stem cell marker LGR5. The results showed that in the intestines of p21-G3TKO mice, even with increased proliferation of cells in the enlarged crypt structure, we could barely observe a few LGR5-positive cells. In the intestines from age-matched G3TKO mice, we observed a few LGR5-positive cells (Figure 3B, LGR5, G3DKO, p21-G3TKO). These data suggested that p21 deficiency might increase the turnover rate of intestinal epithelia and diminish the stem cell reservoir. However, in the background of G5DKO, p16 deficiency rescued the stem cell reservoir and rescued cell proliferation (Figure 3B, LGR5, G5DKO, p16-G5TKO).

### The Telomere Maintenance Mechanism Is Involved in p16 Function but Not p21 Function

**FIGURE 4:**
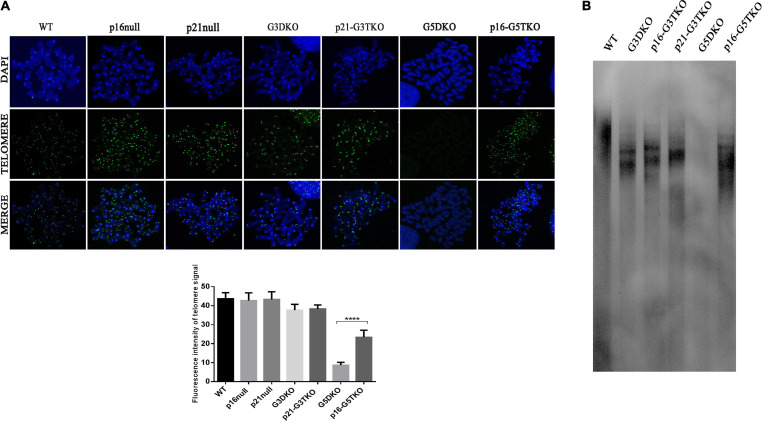
The telomere length of MEFs with different genotypes measured by FISH **(A)** and TRF-Southern blot **(B)**. Quantification of telomere signal and statistical analysis. ****p < 0.001. p21-G3TKO did not affect telomere length, while p16-G5TKO rescued telomere length from attrition.

Telomere maintenance is essential for cellular proliferation and prevents the occurrence of premature aging. We thus evaluated telomere length in MEFs derived from WS mice with or without p21 or p16 deficiency by PNA-FISH and TRF-Southern blot. Both data revealed that, as we expected, the G5DKO MEFs showed the weakest signals for the telomeres, suggesting the severe shortening of telomere length. Interestingly, p16 deficiency rescued telomere length very well, possibly through a new mechanism of ALT. To our surprise, p21 deficiency did not affect telomere length, which might sustain the high proliferation of cells we observed above (Figure 4).

### The Cellular DNA Damage Response Contributed to the Distinct Function of p21 and p16 in the Aging Process of Werner Syndrome

To understand the mechanism of aging regulating WS induced by p21 or p16 deficiency, we used MEFs to analyze the cellular pathways associated with cellular senescence, proliferation and apoptosis.

**FIGURE 5:**
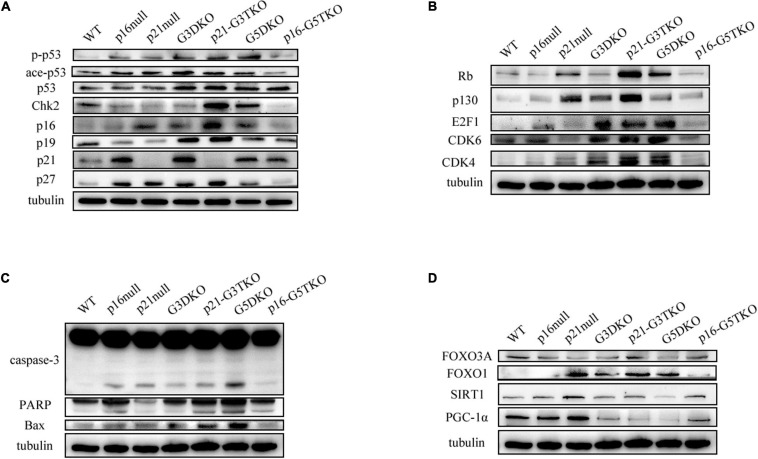
DDR and energy-related pathway analysis by Western blot. **(A)** p53-related DDR pathway. **(B)** Rb-related DDR pathway. **(C)** Apoptosis pathway. **(D)** Energy-related pathway.

The data indicated that the core regulator of the DNA damage response (DDR) pathway, p53, was activated in G3DKO, p21-G3TKO, and G5DKO MEFs at both the phosphorylation and acetylation levels. The upstream regulator of p53, Chk2, was also upregulated, especially in p21-G3TKO MEFs, suggesting the high activation of DDR pathways in p21-G3TKO MEFs (Figure 5A). However, due to p21 deficiency, the direct downstream of p53 DDR was deficient, but the regulators with similar functions, such as p27, p16, p19^ARF^ (p19), the Rb pathway regulators Rb, and p130, were all upregulated in p21-G3TKO MEFs. Interestingly, the proliferation promoters E2F1, CDK6 and CDK4 were also upregulated (Figures 5A,B). Together, these data supported the idea that p21 deficiency in the G3DKO background accelerated cellular proliferation even with a high level of DDR. On the other hand, p16 deficiency in the G5DKO background rescued the high level of DDR derived from G5DKO and did not activate inhibitors such as p53, p19, p27, Rb, p130 or promoters such as E2F1, CDK6, and CDK4 (Figures 5A,B).

Consistent with this, the apoptosis pathway was highly activated in G5DKO, but only slightly elevated in p21-G3TKO, as indicated by increased caspase 3 and PARP cleavage (Figure 5C).

We tried to detect the regulation of energy metabolism-related proteins by p21 or p16 deficiency. We found that FOXO3 did not change much in MEFs with different genotypes. Interestingly, FOXO1 was upregulated by either p21 deficiency (Figure 5D, p21 null) or telomere dysfunction derived from WS (Figure 5D, G3DKO, G5DKO) or both (Figure 5D, p21-G3TKO). p16 deficiency attenuated this upregulation (Figure 5D, p16-G5TKO), suggesting that the upregulation of FOXO1 contributed to the accelerated aging phenotypes of p21-G3TKO. We also observed the downregulation of SIRT1 in G5DKO, which were reversed in p16-G5TKO. PGC1α was downregulated in G3DKO, p21-G3TKO, and G5DKO MEFs but was rescued in p16-G5TKO MEFs (Figure 5D). Together, these data suggest that energy metabolism regulation could be essential for preventing aging; however, the function and crosstalk between key regulators could be very complicated.

## Discussion

The premature aging process of WS is known to be accompanied by tumorigenesis and might be caused by chromosomal aberrations derived from telomere dysfunction (Chang et al., 2004). We observed that the senescent WS MEFs could spontaneously delete *Ink4a* locus chromosomal DNA and became immortalized, during this process, the Trp53 genes maintained wild type status, and the cells could not form tumors in SCID mice (Wu et al., 2012). We speculated that this type of status indicates tumor-free longevity and investigated this *in vivo*.

On the other hand, we also observed that some senescent WS MEFs could spontaneously mutate the Trp53 gene and thus diminished p21 function, and these cells were able to form ALT tumors in SCID mice. The exogenous expression of p21 in these cells rebuilt the senescence status (Jia et al., 2012; Si et al., 2018). These data were consistent with previous findings and suggested that the p53 or p21 functional loss could also rescue cellular senescence, but with the price of tumorigenesis (Ferbeyre and Lowe, 2002). Thus, we speculated that this type of status was tumor-prone.

In previous studies, p16 has been found to be essential in modifying the stemness and capacity of stem cells, and p16 deficiency has been found to be able to rescue aging phenotypes and increase stem cell capacity in aged mice (Janzen et al., 2006; Krishnamurthy et al., 2006; Molofsky et al., 2006; Diekman et al., 2018). p16 has also been found to be able to prevent the aging process and elongate lifespan in several prematurely aging mouse models. In mutant mice homozygous for a hypomorphic allele of the (alpha)-klotho aging-suppressor gene, accelerated aging phenotypes were rescued by p16 deficiency, possibly through the restoration of (alpha)-klotho expression (Sato et al., 2015). In the progeroid mouse model derived from the aberration of the mitosis regulator BubR1, p16 deficiency attenuated both cellular senescence and premature aging in skeletal muscle and fat tissues; thus, the clearance of p16-positive senescent cells could delay aging-associated disorders in BubR1-insufficient mice (Baker et al., 2008, 2011). Surprisingly, the same study also revealed an opposing role of p19^ARF^ in modulating aging phenotypes in BubR1-insufficient mice. Rather than delayed aging, p19 deficiency accelerated aging phenotypes in skeletal muscle and fat tissues, indicating that p19 provides anti-aging activity (Baker et al., 2008).

In a study using mice with telomere dysfunction caused by knocking out the telomerase RNA component TERC (*mTerc*^–/–^), the loss of the *Ink4a* locus, encoding both p16Ink4a and p19Arf, did not attenuate the degenerative phenotypes in late-generation mice. Interestingly, the tumor onset in *mTerc*^–/–^ mice was even delayed by the loss of the *Ink4a* locus (Khoo et al., 2007).

Furthermore, a study conducted in Pot1b knockout mice provided a new idea about the role of p16 or p21 in regulating the aging process. In this study, knocking out p16 resulted in tissue damage and dysfunction of proliferative tissues, such as the hematopoietic system, intestine, and testis. On the other hand, knocking out p21 in Pot1b-deficient mice could rescue the aging phenotypes and elongate the lifespan. This study also showed that p16 deficiency could activate ATR-induced DDR and accelerate the aging process (Wang et al., 2013).

Here, in the background of WS, our data revealed that p21 deficiency accelerated the progress of progeroid phenotypes, such as the degeneration of bone, testis, and intestine. Interestingly, our data indicated that p21 deficiency resulted in a dramatic increase in cellular senescence, apoptosis, and proliferation in these tissues. In addition to the decreased stem cell reservoir in the case of p21 deficiency, these data suggested that p21 deficiency promoted the turnover of tissue cells, which resulted in the exhaustion of the intestinal stem cell reservoir. These data suggest that p21 protected the stem cell reservoir by regulating cellular proliferation and turnover at a proper rate. The expression regulation of DDR pathway-related proteins also indicated that p27, p16, p19, Rb, and p130 were all upregulated in p21-G3TKO MEFs, suggesting that p21 loss in WS activated fairly severe DDR responses and might have caused an abnormal increase in tissue repair.

In the case of p16 deficiency, the aging phenotypes of G5DKO were dramatically rescued, showing reduced cellular senescence and apoptosis. On the other hand, the cellular proliferation was increased in the case of p16 deficiency, as well as the stem cell reservoir. These data indicated that p16 promoted cellular senescence by inhibiting cellular proliferation and that p16 deficiency released this barrier signal without causing severe DDR.

These data are consistent with the previous finding for the opposing role of p19 or p16 in BubR1-deficient mice, suggesting a similar function of p21 and p19 in modulating the aging process (Baker et al., 2008). As the key mediator of p53 function, our data are also consistent with a previous study showing that the regulated overexpression of p53 could promote longevity (Matheu et al., 2007). We are puzzled by the reverse effect of p21 and p16 in WS mice with previously reported Pot1b-deficient mice (Wang et al., 2013). We hypothesize that p21 and p16 might play different roles in different genetic backgrounds, which might result in different amounts of background DNA damage. In other words, the extent of DNA damage determines the role of p21 or p16 in the aging process. Further investigation is needed to verify this idea.

Interestingly, the data also suggested that p21 loss did not affect the telomere attrition rate, while p16 deficiency attenuated telomere attrition and rescued the stem cell reservoir. These data suggest a new role for p16 in inhibiting telomere lengthening via the ALT mechanism.

We were expecting a tumorigenesis model when we crossed *p21*^–/–^ mice with WS mice; to our surprise, we obtained an accelerated progeroid mouse model. We did not observe any tumorigenesis in p21-TKO mice. We are not sure whether this is because the short lifespan masks the tumorigenesis phenotype, or the accelerated aging inhibited tumorigenesis.

It has been shown that telomere dysfunction induces metabolic and mitochondrial compromise and suppresses the PGC-1α network (Sahin et al., 2011). We also found that PGC-1α is downregulated in late generation WS mice, G3DKO and G5DKO MEFs, as well as in p21-G5TKO MEFs. On the other hand, p16 deficiency could rescue the expression of PGC-1α, which could contribute to the anti-aging effect of p16. However, we also observed the upregulation of SIRT1 in G5DKO and p21-G5TKO MEFs. These data suggested that the regulation of energy metabolism could be quite complicated, and further research needs to be conducted to understand the systems network.

In summary, our data revealed that p21 deficiency accelerated the aging process in WS, shortened mouse lifespan, and accelerated the degeneration of bone, testis, and intestinal tissues. p21 deficiency resulted in a dramatic increase in DNA damage responses, cellular senescence, apoptosis, and proliferation, while a decreased stem cell reservoir suggested that p21 deficiency promoted the turnover of tissue cells and exhaustion of stem cell reservoirs. In the case of p16 deficiency, the aging phenotypes of WS were dramatically rescued, showing reduced cellular senescence, apoptosis, increased telomere length, cellular proliferation, and the stem cell reservoir.

These data suggest that p21 protected the stem cell reservoir by regulating cellular proliferation and turnover at a proper rate and that p21 loss in WS activated fairly severe DDR responses, which might have caused an abnormal increase in tissue repair. On the other hand, p16 promoted cellular senescence by inhibiting cellular proliferation, and p16 deficiency released this barrier signal without causing severe DDR.

## Materials and Methods

### Mice

The *p21*^–/–^ mice were kindly provided by Dr. Tyler Jacks from MIT. The WS (*mTR*^–/–^*Wrn*^–/–^) and *p16*^–/–^ mice were kindly provided by Dr. Sandy Chang from Yale University and Dr. Ronald A. Depinho from MD Anderson Cancer Center, respectively.

We crossed the WS mice (double knock out, DKO) with *p21*^–/–^ or *p16*^–/–^ mice and obtained the first generation (G1) triple knockout mice *mTer*^–/–^*Wrn*^–/–^*p21*^–/–^ (p21-TKO) or *mTer*^–/–^*Wrn*^–/–^*p16*^–/–^ (p16-TKO). The mice were then inbred generation by generation and G2, G3, G4, G5 TKO mice were obtained. The WS mice and wild type (WT) mice were used as controls. All experiments were carried out with the approval of the Animal Care and Use Committee of the Kunming University of Science & Technology (approval ID: M2012-020) in accordance with the guidelines of the Association for Assessment and Accreditation of Laboratory Animal Care.

### MEF Cells

Mouse embryo fibroblast cells with different genotypes were harvested in E13.5 days and cultured in Dulbecco’s Modified Eagle Medium (DMEM) with 10% fetal bovine serum (FBS) at 37°C with 5% CO_2_ and 3% O_2_. To maintain the characteristics of their origin, only the early passages (≤passage 5) of MEF cells were used for experiments.

### Pathology Analysis

The tissue samples were fixed in 4% paraformaldehyde (PFA) at 4°C overnight and then alcohol-dehydrated and paraffin-embedded. The paraffin-embedded tissue blocks were sectioned into 4 μm slices for later experiments. For hematoxylin-eosin (HE) staining, the tissue sections were deparaffinized and rehydrated, and HE staining was applied. For the immunohistochemistry assay, after the tissue sections were deparaffinized and rehydrated, the endogenous peroxidase activity was blocked by treatment with 3% hydrogen peroxide in methanol. After steaming in 10 mM citrate buffer (pH 6) for antigen retrieval, the slides were blocked with goat serum and incubated in primary antibody in blocking solution at 4°C overnight. Secondary antibodies in blocking buffer were applied for 2 h at room temperature. After washing three times in 1x PBS for 10 min, the slides were incubated with 3,3-diaminobenzidin at room temperature in the dark for 3 min. For the TUNEL assay, the slides were deparaffinized, rehydrated, permeabilized, and applied to the TdT activity test with a TUNEL assay kit (Roche).

For BrdU staining, BrdU was intraperitoneally injected twice into the mouse at 24 and 6 h (200 μg/time) before sample collection. The tissues were then collected and subjected to IHC. The following primary antibodies were used: anti-LGR5 (1:25, Abcam) and anti-BrdU (1:10, BD Biosciences).

### Western Blotting

Cells were lysed in RIPA buffer containing Protease Inhibitor Cocktail (Roche). Sample proteins (20 μg) were separated by SDS-PAGE and then transferred to PVDF membranes. After blocking in 10% nonfat milk for 1 h at room temperature, membranes were incubated with primary antibodies overnight at 4°C or 2 h at room temperature. The membranes were then incubated with horseradish peroxidase-labeled secondary antibodies and visualized with ECL. The following primary antibodies were used: anti-p53 (1:500, CST), anti-phosphorylated p53 (Ser15) (1:500, CST), anti-acetylated p53 (1:800, CST), anti-Chk2 (1:500, BD Biosciences), anti-p21 (1:500, BD Biosciences), anti-p27 (1:1800, BD Biosciences), anti-p16 (1:1,200, SAB), anti-p19 (1:600, Thermo), anti-Rb (1:500, BD Biosciences), anti-p130 (1:800, Abcam), anti-Caspase 3 (1:800, CST), anti-PARP (1:800, CST), anti-Bax (1:500, CST), anti-CDK4 (1:800, CST), anti-CDK6 (1:800, CST), anti-E2F1 (1:500, BD Biosciences), anti-Foxo1 (1:500, Millipore), anti-Foxo3a (1:500, Millipore), anti-Sirt1 (1:800, CST), and anti-γ-tubulin (1:8000, Millipore).

### SA-β-Gal Staining

Tissues were freshly collected and embedded in OCT, and the OCT blocks were frozen and cryostat sectioned into 5 μm slices for later experiments. For SA-β-Gal staining, the slides were fixed for 3 min (room temperature) in 4% PFA, followed by 1x PBS washing three times. Fixed tissue slides were stained with fresh staining solution for SA-β-galactosidase activity at 37°C for 4 h, as described previously (Dimri et al., 1995).

### Telomere FISH and TRF-Southern Blot Assay

Mouse embryo fibroblasts with different genotypes were used to perform telomere FISH and TRF-Southern blot assays. For telomere FISH, the metaphases of MEFs were prepared and subjected to FISH analysis using a telomere PNA probe (FITC-TTAGGG, PANAGENE).

For the TRF-Southern blot assay, 40 μg of genomic DNA was digested by Hinf I and Rsa I and resolved by pulsed field gel electrophoresis (PFGE). The DNA was transferred to a nylon membrane, and the hybridization was then performed with a telomere length assay kit (TeloTAGGG, Roche) according to the manufacturer’s instructions.

### Bone Density Measurement

Mouse bone density was measured by MicroCT. Prior to MicroCT scanning, the mice were sacrificed, and the femur samples were collected and fixed in 10% neutral buffered formalin and then transferred to 70% ethanol. Excised femurs were scanned on a Latheta LCT-200 scanner (Hitachi-Aloka, Tokyo, Japan) using high resolutions.

## Data Availability Statement

All datasets presented in this study are included in the article/supplementary material.

## Ethics Statement

The animal study was reviewed and approved by the Animal Care and Use Committee of the Kunming University of Science & Technology (approval ID: M2012-020). Written informed consent was obtained from the owners for the participation of their animals in this study.

## Author Contributions

All authors listed have made a substantial, direct and intellectual contribution to the work, and approved it for publication. YZ and CS contributed equally to this work.

## Conflict of Interest

The authors declare that the research was conducted in the absence of any commercial or financial relationships that could be construed as a potential conflict of interest.
